# Genomic insights highlight antimicrobial potential of *Micromonospora* sp. PTRAS2

**DOI:** 10.1128/mra.01306-25

**Published:** 2026-01-28

**Authors:** Pankaj Mandal, Trisha Roy, Rittick Mondal, Arka Pratim Chakraborty, Priyambada Singh, Amit Ghati, Arnab Sen, Abdul Sadat, Amit Kumar Mandal

**Affiliations:** 1 Department of Sericulture, Raiganj University 561306, Raiganj, West Bengal, India; 2 Department of Microbiology, Kalinga University 653278, Raipur, Chhattisgarh, India; 3 Department of Botany, Raiganj University 561306, Raiganj, West Bengal, India; 4 Department of Microbiology, Barrackpore Rastraguru Surendranath College 207565 , Kolkata, West Bengal, India; 5 Bioinformatics Facility Center, Department of Botany, University of North Bengal 30189, Darjeeling, West Bengal, India; DOE Joint Genome Institute, Berkeley, California, USA

**Keywords:** genomics

## Abstract

*Micromonospora* sp. strain PTRAS2, a gram-positive actinobacterium isolated from mulberry rhizospheric soil in Raiganj, India, has a 7.6 Mb genome (G + C 73.7%). Genome analysis revealed multiple biosynthetic gene clusters for antimicrobial and bioactive compounds, indicating its potential as a source of novel natural products.

## ANNOUNCEMENT

The genus *Micromonospora* comprises gram-positive, aerobic, filamentous actinobacteria that typically form orange colonies, which often turn red, brown, or black upon sporulation (1). Members are widely distributed in soil and plant-associated niches and are renowned for producing structurally diverse secondary metabolites, including antibiotics and hydrolytic enzymes (2, 3). Their extensive biosynthetic gene clusters (BGCs) repertoire makes *Micromonospora* species prime candidates for natural product discovery and biotechnological research.

*Micromonospora* sp. strain PTRAS2 was isolated from the rhizospheric soil of a mulberry plant (GPS coordinates: 25.6072°N, 88.1303°E; sampling date: 6 April, 2025; sampling time: 10:30 a.m. IST). Ten grams of soil samples were suspended in 90 mL sterile 0.9% NaCl, serially diluted, and spread on starch casein agar (starch 10.0 g, KNO_3_ 2.0 g, NaCl 2.0 g, K_2_HPO_4_ 2.0 g, MgSO_4_·7H_2_O 0.05 g, CaCO_3_ 0.02 g, FeSO_4_ 0.01 g, casein 0.3 g, agar, 20.0 g, distilled water, 1 L, and pH 7.0), and incubated at 30°C for 4–5 days (3). Single colonies were purified by repeated streaking. A single representative colony obtained after the third passage, showing consistent colony morphology across successive subcultures, was selected for further analysis, maintained for up to 12 generations, and preserved at −80°C in 20% glycerol.

**Fig 1:**
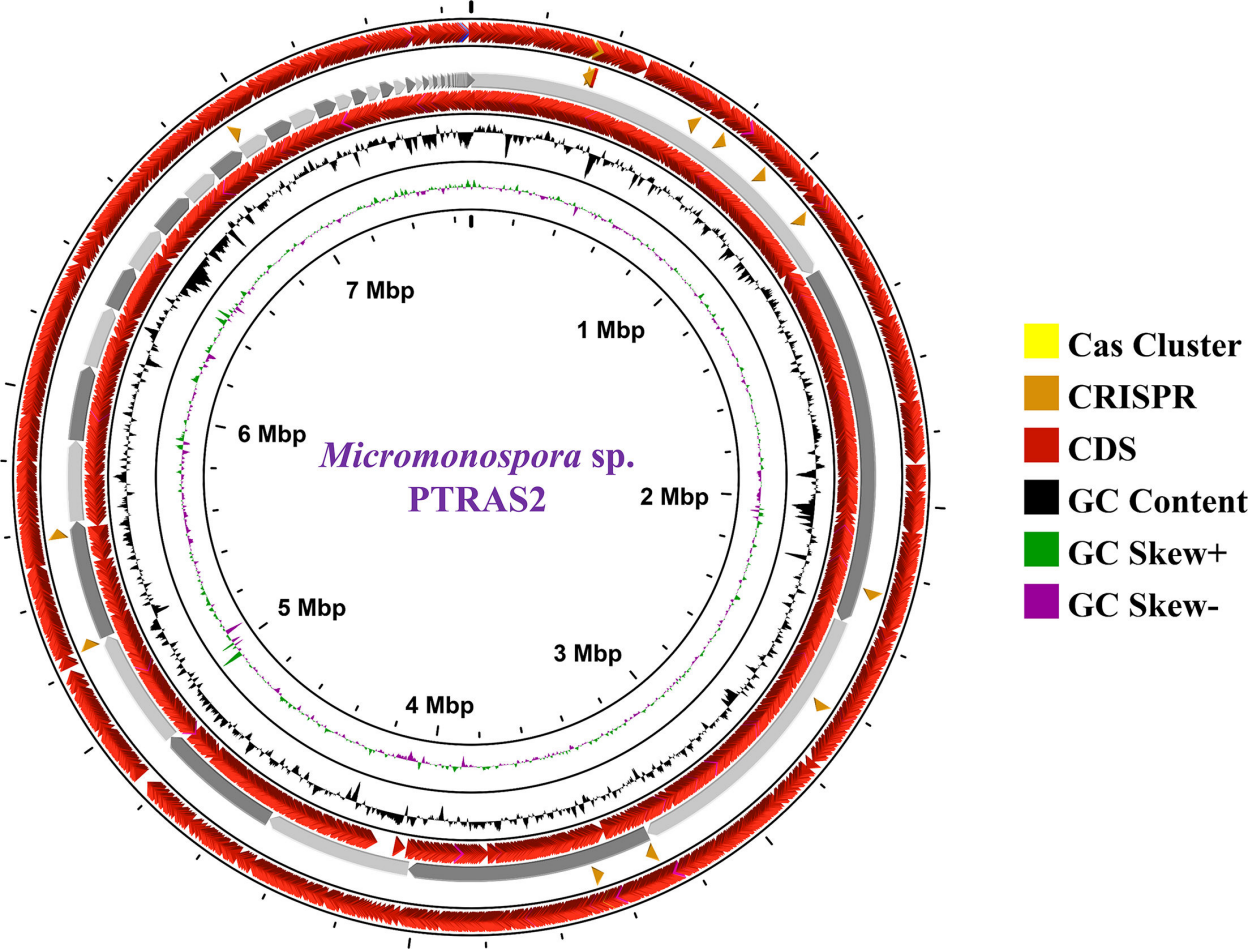
A circular representation of the *Micromonospora* sp. strain PTRAS2 genome generated using CGView. From outside to center, rings 1 and 4 show predicted coding sequences (CDSs) on both the forward and reverse strand; ring 2 shows CRISPR-Cas systems features; ring 3 (backbone) shows contigs; ring 5 shows G+C% content plot, and ring 6 shows GC skew.

Genomic DNA was extracted from this single-colony isolate using the standard phenol-chloroform method (4). Paired-end libraries were prepared using the KAPA HyperPlus Kit (Roche #07962428001) and sequenced on the Illumina NovaSeq 6000 platform. Library quantification and insert size assessment were performed using Qubit 4.0 and LabChip GX Touch. Sequencing generated 29,570,242 reads (2 × 150  bp). Raw reads were quality-checked using FastQC v.0.11.9 and preprocessed with Fastp v.0.23.4 (5, 6) (parameters: *--length_required 50 --correction --trim_poly_g --cut_front --cut_tail --qualified_quality_phred 30 --unqualified_percent_limit 30 --average_qual 30*). Processed reads were *de novo* assembled using Unicycler v.0.4.4 (7). Genome completeness and contamination were estimated using CheckM2 v.1.0.1 (8). Annotation was performed using BV-BRC v3.54.6 (9). All software tools were run using default parameters unless otherwise specified. The final assembly comprised 82 contigs, with an *N*_50_ of 756,057 bp and an *L*_50_ of 4. The draft genome is 7,677,193 bp, with a G + C content of 73.7% and an average sequencing coverage of 577.75×. A total of 6,839 coding sequences were annotated, including 5 rRNA genes and 59 tRNA genes. A circular genome map was generated using CGView (Fig. 1) (10).

Genome mining revealed a diverse array of BGCs, including those for polyketides, nonribosomal peptides, and ribosomally synthesized and post-translationally modified peptides clusters. Several clusters showed high similarity to known biosynthetic systems such as for loseolamycin A1/A2, SapB, and chromomycin A3, suggesting the strain’s potential to produce novel bioactive compounds. Additionally, the genome harbored antimicrobial resistance determinants including *qacG* and *vanW*, and a *rox* homolog from *Streptomyces venezuelae*, suggesting a potential horizontal transfer of rifamycin-modifying elements. A well-defined Type I-E CRISPR-Cas system with multiple CRISPR arrays was also identified, underscoring genomic plasticity, adaptability, and dual strategies for survival via antimicrobial resistance and adaptive immunity.

## Data Availability

The whole-genome shotgun project of Micromonospora sp. strain PTRAS2 has been deposited at NCBI under GenBank accession number JBSWBU000000000. The genome assembly accession number is GCA_054052245.1. The BioSample and BioProject accession numbers are SAMN52635495 and PRJNA1344308, respectively. The raw data are available in the Sequence Read Archive (SRA) under accession number SRR35891012.
